# Complete genome sequence of *Rhodococcus qingshengii* strain A3-8

**DOI:** 10.1128/mra.00418-26

**Published:** 2026-09-17

**Authors:** Nobuhiro Takahashi, Ryota Moriuchi, Hideo Dohra, Hiroyuki Futamata

**Affiliations:** 1 Graduate School of Science and Technology, Shizuoka University 13058, Shizuoka, Japan; 2 Shizuoka Instrumental Analysis Center, Shizuoka University 13058, Shizuoka, Japan; 3 Research Institute of Green Science and Technology, Shizuoka University 13058, Shizuoka, Japan; 4 Department of Science, Graduate School of Integrated Science and Technology, Shizuoka University 13058, Shizuoka, Japan; 5 Department of Engineering, Graduate School of Integrated Science and Technology, Shizuoka University 13058, Shizuoka, Japan; Indiana University Bloomington, Bloomington, Indiana, USA

**Keywords:** *Rhodococcus*, genome analysis

## Abstract

A chemostat culture was constructed with phenol and forest soil as an inoculum. We report the complete genome sequence of *Rhodococcus qingshengii* strain A3-8, which was isolated from the culture. The genome consists of a chromosome (6,436,695 bp) and a linear plasmid pA38 (257,365 bp).

## ANNOUNCEMENT

*Rhodococcus qingshengii* strain A3-8, a gram-positive bacterium, was aerobically isolated from a phenol-fed chemostat culture inoculated with forest soil (34°42′45.0″N, 137°41′14.6″E) using a dilute plating method with MP medium (1, 2) containing the culture supernatant on day 253. The complete genome of strain A3-8 was sequenced to investigate the genomic basis of long-term survival strategy. Strain A3-8 was incubated in 24 mL Luria broth with 4% glycine for 2 days at 25°C. DNA was extracted by previously described (3, 4). We determined the complete genome sequence of DNA that was extracted from the same inoculum using PacBio Sequel II (long reads) and Illumina MiSeq (short reads) sequencer. A PacBio sequencing library was prepared using the SMRTbell prep kit 3.0 (Pacific Biosciences) and sequenced by the Sequel systems (Macrogen, Inc., Seoul, South Korea). An Illumina sequencing library was constructed using the TruSeq Nano DNA library preparation kit (Illumina) and sequenced on the Illumina MiSeq system. PacBio HiFi reads were filtered (read lengths, ≥7,000 bp) using SeqKit v. 0.8.0 (5) and assembled by Flye v. 2.8.3 (6) with—plasmid option. One circular contig and one linear contig were obtained. The circular contig was rotated to start with the *dnaA* gene using Circlator v. 1.1.1 (7). Illumina reads were cleaned (quality scores, ≥15; read lengths, ≥150 bp) using Trimmomatic v. 0.39 (8), and the resulting high-quality reads were aligned by BWA-MEM v. 0.7.15 (9) and corrected bases by Pilon v. 1.23 (10). The gene prediction and annotation were performed using DFAST-core v. 1.2.18 (11) with an in-house database containing 585 genome sequences of the genus *Rhodococcus* deposited in the National Center for Biotechnology Information (NCBI) RefSeq database (21 April 2023). GeneMarkS2 v. 1.14_1.25 (12) and MetaGeneAnnotator version 2008/08/19 (13), RNAmmer v. 1.2 (14), and tRNAscan-SE v. 2.0.5 (15) were used for the prediction of protein-coding sequences, ribosomal RNA (rRNA) genes, and transfer RNA (tRNA) genes, respectively. Default parameters were used for all software unless otherwise specified. Information on the PacBio and Illumina reads is summarized in Table 1. The genome consists of a circular chromosome (6,436,695 bp with a GC content of 62.5%) and a linear plasmid (pA38 [257,365 bp] with a GC content of 60.6%). Based on a pLAST analysis (16), pA38 was identified as a *Rhodococcus* plasmid, exhibiting the highest score of 0.97 against a circular plasmid from *Rhodococcus* sp. AD45. Furthermore, pA38 possesses telomeric invertron-like terminal inverted repeats at both ends, indicating its linear topology (17, 18). The genome has 6,150 protein-coding sequences, 5 sets of rRNA genes, and 59 tRNA genes. Average nucleotide identity based on BLAST+ (ANIb) and digital DNA-DNA hybridization (dDDH) values were calculated via JSpeciesWS (https://jspecies.ribohost.com/jspeciesws/) (19) and Genome-to-Genome Distance Calculator 3.0 (https://ggdc.dsmz.de/ggdc.php) (20), respectively, using genome sequences from 17 selected type strains of *Rhodococcus* species available in the NCBI RefSeq database. The results indicated that strain A3-8 was closely related to *R. qingshengii* djl-6^T^ (accession number CP096563) with an ANIb value of 97.8% and a dDDH value of 85.2%, suggesting that strain A3-8 could be recognized as *R. qingshengii*.

**TABLE 1:** Summary of reads generated by Illumina and PacBio sequencing

Parameter	Data for sequencing with	
	PacBio Sequel II	Illumina MiSeq
Raw reads		
No. of reads	131,803	1,407,864
Total size (bp)	1,255,437,342	420,596,337
N_50_ (bp)	10,156	
Filtered reads		
No. of reads	109,320	1,293,820
Total size (bp)	1,129,423,741	331,060,406
N_50_ (bp)	10,492	
Mean coverage (×)^*a*^	175.5	51.4
Accession no.	DRR495761	DRR495806

^*a*^ Total size of filtered reads (bp)/genome size of A3-8 (bp).

## Data Availability

The complete sequences of the chromosome and plasmid pA38 have been deposited in the DDBJ/EMBL/GenBank databases under the accession numbers AP028674 (chromosome) and AP028675 (plasmid pA38), respectively. The raw PacBio and Illumina sequencing reads are available under DRR495761 and DRR495806. All project data are available under BioProject accession number PRJDB16326.

## References

[1] Watanabe K, Yamamoto S, Hino S, Harayama S. 1998. Population dynamics of phenol-degrading bacteria in activated sludge determined by gyrB-targeted quantitative PCR. Appl Environ Microbiol 64:1203–1209. doi:10.1128/AEM.64.4.1203-1209.1998 PMC106130 9546154

[2] Watanabe K, Hino S, Onodera K, Kajie S-I, Takahashi N. 1996. Diversity in kinetics of bacterial phenol-oxygenating activity. J Ferment Bioeng 81:560–563. doi:10.1016/0922-338X(96)81481-4

[3] Watanabe K, Teramoto M, Futamata H, Harayama S. 1998. Molecular detection, isolation, AND physiological characterization of functionally dominant phenol-degrading bacteria in activated sludge. Appl Environ Microbiol 64:4396–4402. doi:10.1128/AEM.64.11.4396-4402.1998 PMC106659 9797297

[4] Nahar A, Baker AL, Nichols DS, Bowman JP, Britz ML. 2021. Benchmarking DNA extraction methods for phylogenomic analysis of sub-antarctic *Rhodococcus* and *Williamsia* species. Microorganisms 9:1253. doi:10.3390/microorganisms9061253 PMC8227252 34207615

[5] Shen W, Le S, Li Y, Hu F. 2016. SeqKit: a cross-platform and ultrafast toolkit for FASTA/Q file manipulation. PLoS One 11:e0163962. doi:10.1371/journal.pone.0163962 PMC5051824 27706213

[6] Kolmogorov M, Yuan J, Lin Y, Pevzner PA. 2019. Assembly of long, error-prone reads using repeat graphs. Nat Biotechnol 37:540–546. doi:10.1038/s41587-019-0072-8 30936562

[7] Hunt M, Silva ND, Otto TD, Parkhill J, Keane JA, Harris SR. 2015. Circlator: automated circularization of genome assemblies using long sequencing reads. Genome Biol 16:294. doi:10.1186/s13059-015-0849-0 PMC4699355 26714481

[8] Bolger AM, Lohse M, Usadel B. 2014. Trimmomatic: a flexible trimmer for Illumina sequence data. Bioinformatics 30:2114–2120. doi:10.1093/bioinformatics/btu170 PMC4103590 24695404

[9] Li H. 2013. Aligning sequence reads, clone sequences and assembly contigs with BWA-MEM.

[10] Walker BJ, Abeel T, Shea T, Priest M, Abouelliel A, Sakthikumar S, Cuomo CA, Zeng Q, Wortman J, Young SK, et al.. 2014. Pilon: an integrated tool for comprehensive microbial variant detection and genome assembly improvement. PLoS One 9:e112963. doi:10.1371/journal.pone.0112963 PMC4237348 25409509

[11] Tanizawa Y, Fujisawa T, Nakamura Y. 2018. DFAST: a flexible prokaryotic genome annotation pipeline for faster genome publication. Bioinformatics 34:1037–1039. doi:10.1093/bioinformatics/btx713 PMC5860143 29106469

[12] Lomsadze A, Gemayel K, Tang S, Borodovsky M. 2018. Modeling leaderless transcription and atypical genes results in more accurate gene prediction in prokaryotes. Genome Res 28:1079–1089. doi:10.1101/gr.230615.117 PMC6028130 29773659

[13] Noguchi H, Taniguchi T, Itoh T. 2008. MetaGeneAnnotator: detecting species-specific patterns of ribosomal binding site for precise gene prediction in anonymous prokaryotic and phage genomes. DNA Res 15:387–396. doi:10.1093/dnares/dsn027 PMC2608843 18940874

[14] Lagesen K, Hallin P, Rødland EA, Staerfeldt H-H, Rognes T, Ussery DW. 2007. RNAmmer: consistent and rapid annotation of ribosomal RNA genes. Nucleic Acids Res 35:3100–3108. doi:10.1093/nar/gkm160 PMC1888812 17452365

[15] Chan PP, Lin BY, Mak AJ, Lowe TM. 2021. tRNAscan-SE 2.0: improved detection and functional classification of transfer RNA genes. Nucleic Acids Res 49:9077–9096. doi:10.1093/nar/gkab688 PMC8450103 34417604

[16] Krakowski K, Orlowska M, Kaminski K, Bartosik D, Dunin-Horkawicz S. 2025. pLAST-a tool for rapid comparison and classification of bacterial plasmid sequences. bioRxiv. doi:10.1101/2025.11.27.689987 PMC13481667 42525290

[17] Sekine M, Tanikawa S, Omata S, Saito M, Fujisawa T, Tsukatani N, Tajima T, Sekigawa T, Kosugi H, Matsuo Y, et al.. 2006. Sequence analysis of three plasmids harboured in *Rhodococcus erythropolis* strain PR4. Environ Microbiol 8:334–346. doi:10.1111/j.1462-2920.2005.00899.x 16423019

[18] Francis I, De Keyser A, De Backer P, Simón-Mateo C, Kalkus J, Pertry I, Ardiles-Diaz W, De Rycke R, Vandeputte OM, El Jaziri M, et al.. 2012. pFiD188, the linear virulence plasmid of *Rhodococcus fascians* D188. Mol Plant Microbe Interact 25:637–647. doi:10.1094/MPMI-08-11-0215 22482837

[19] Richter M, Rosselló-Móra R, Oliver Glöckner F, Peplies J. 2016. JSpeciesWS: a web server for prokaryotic species circumscription based on pairwise genome comparison. Bioinformatics 32:929–931. doi:10.1093/bioinformatics/btv681 PMC5939971 26576653

[20] Meier-Kolthoff JP, Carbasse JS, Peinado-Olarte RL, Göker M. 2022. TYGS and LPSN: a database tandem for fast and reliable genome-based classification and nomenclature of prokaryotes. Nucleic Acids Res 50:D801–D807. doi:10.1093/nar/gkab902 PMC8728197 34634793

